# Supplementary material for: Tumorspheres as In Vitro Model for Identifying Predictive Chemoresistance and Tumor Aggressiveness Biomarkers in Breast and Colorectal Cancer
Source: Biology (Basel). 2024 Sep 15;13(9):724. doi: 10.3390/biology13090724 (PMC11429065; doi:10.3390/biology13090724)
Supplement: Supplementary file 1 [file biology-13-00724-s001.zip › biology-3164594-supplementary.pdf]

Supplementary Data

Table S1.- Primer sequences for mRNA expression analysis using real-time quantitative PCR.

GENE	FORWARD PRIMER (5'–3') / REVERSE PRIMER (5'–3')	Tº An. (°C)
TBP	TTGGGTTTTCCAGCTAAGTTCT	61
	CCAGGAAATAACTCTGGCTCA	
YWHAZ	CCGTTACTTGGCTGAGGTTG	60
	TGCTTGTTGTGACTGATCGAC	
HMBS	TGTGGTGGGAACCAGCTC	60
	TGTTGAGGTTTCCCCGAAT	
PPIA	ATGCTGGACCCAACACAAAT	60
	TCTTTCACTTTGCCAAACACC	
18S	GGACACGGACAGGATTGACA	61
	ACCCACGGAATCGAGAAAGA	
B2M	TTTCATCCATCCGACATTGA	54
	CGGCAGGCATACTCATCTTT	
ACTB	GCCCAGAGCAAGAGAGGCAT	65
	GCCCATCTCTTGCTCGAAGT	
CXCL8	GGCACAAACTTTCAGAGACAGCAG	67
	GTTTCTTCCTGGCTCTTGTCCTAG	
IL6	CAGGGGTGGTTATTGCATCT	60
	AGGAGACTTGCCTGGTGAAA	
IL6R	TGGGAGGTGGAGAAGAGAGA	61
	AGGACCTCAGGTGAGAAGGCA	
NFKB	CCTGGATGACTCTTGGGAAA	58
	TCAGCCAGCTGTTTCATGTC	
PPARG	GAGCCCAAGTTTGAGTTTGC	61
	CTG TGAGGACTCAGGGTGGT	
TGFB	TCCTGGCGATACCTCAGCAA	60
	CGGTAGTGAACCCGTTGATG	

Tº An.: annealing temperature.
